# Ductal Breast Carcinoma In Situ: Mammographic Features and Its Relation to Prognosis and Tumour Biology in a Population Based Cohort

**DOI:** 10.1155/2017/4351319

**Published:** 2017-02-14

**Authors:** Wenjing Zhou, Thomas Sollie, Tibor Tot, Carl Blomqvist, Shahin Abdsaleh, Göran Liljegren, Fredrik Wärnberg

**Affiliations:** ^1^Department of Surgical Sciences, Uppsala University, Uppsala Academic Hospital, Uppsala, Sweden; ^2^Department of Pathology, Örebro University, Örebro, Sweden; ^3^Department of Pathology, Falun Central Hospital, Falun, Sweden; ^4^Department of Oncology, Helsinki University Central Hospital, Helsinki, Finland; ^5^Department of Radiology, Oncology and Radiation Science, Uppsala University, Uppsala, Sweden; ^6^Department of Surgery, Örebro University, Örebro, Sweden

## Abstract

Casting-type calcifications and a histopathological picture with cancer-filled duct-like structures have been presented as breast cancer with neoductgenesis. We correlated mammographic features and histopathological neoductgenesis with prognosis in a DCIS cohort with long follow-up. Mammographic features were classified into seven groups according to Tabár. Histopathological neoductgenesis was defined by concentration of ducts, lymphocyte infiltration, and periductal fibrosis. Endpoints were ipsilateral (IBE) in situ and invasive events. Casting-type calcifications and neoductgenesis were both related to high nuclear grade, ER- and PR-negativity, and HER2 overexpression but not to each other. Casting-type calcifications and neoductgenesis were both related to a nonsignificant lower risk of invasive IBE, HR 0.38 (0.13–1.08) and 0.82 (0.29–2.27), respectively, and the HR of an in situ IBE was 0.90 (0.41–1.95) and 1.60 (0.75–3.39), respectively. Casting-type calcifications could not be related to a worse prognosis in DCIS. We cannot explain why a more aggressive phenotype of DCIS did not correspond to a worse prognosis. Further studies on how the progression from in situ to invasive carcinoma is driven are needed.

## 1. Background

In early breast cancers, a subgroup with characteristic “casting type” calcifications on the mammograms with or without an associated tumour mass has been described. These casting type calcifications are sometimes present in large numbers and unnaturally tightly packed, often filling an entire lobe. The corresponding histopathological picture has been described by Tabar et al. [1, 2] as cancer-filled duct-like structures associated with periductal lymphocytic infiltration and a periductal desmoplastic reaction. This duct-forming process does not fit in the classical description of invasive or in situ breast cancer and was presented by the term “breast cancer with neoductgenesis” [1, 2]. Tenascin-C (Tn-C) overexpression was also related to this mammographic and histopathological picture. In a recent paper, we tried to define and quantify histopathological criteria for the proposed diagnosis of breast cancer with neoductgenesis [3]. We showed that mammographic calcifications (crushed stone-like and casting type calcifications together) were related to the combination of concentration of ducts, lymphocyte infiltration, and fibrosis in cases with ductal breast carcinoma in situ (DCIS), with or without an invasive component. These three histopathological features in combination were also related to a more aggressive tumour phenotype, especially HER2 overexpression [3].

DCIS is often detected by mammography and cases with malignant microcalcifications on the mammograms often reveal DCIS on a preoperative core biopsy [4, 5]. Women with DCIS have an excellent prognosis as a group but some cases do recur as invasive cancer or even as generalized disease. Treatment decisions could be influenced by identifying a group of DCIS with a significantly higher risk of recurrence or even risk of breast cancer death. And we could possibly avoid radiotherapy (RT) or mastectomy in many cases if we were able to distinguish those with a low risk of progression to invasive cancer.

In this study, the aim was to correlate mammographic features and especially casting type calcifications with prognosis in a large cohort of women with primary DCIS and a long follow-up. We also correlated the mammographic findings with earlier defined histopathological criteria for breast cancer with neoductgenesis.

## 2. Material and Methods

### 2.1. Patients

Women diagnosed with a primary DCIS between 1986 and 2004 from Uppland and Västmanland counties in Sweden were all included (*n* = 458). The baseline clinical and histopathological characteristics for this population based cohort have been presented earlier [6]. Follow-up was complete up to December 15, 2013. The study was approved by the ethics committee of Uppsala University, Sweden (Dnr. 2005:118 and 2007:315). No informed consent was needed.

### 2.2. Histopathology and Immunohistochemistry

All cases were histopathologically reexamined before the construction of tissue microarrays (tma) and histopathological grade of DCIS was classified according to the European Organization for Research and Treatment of Cancer (EORTC) system. Tma data was used for ER, PR, and Ki67 by immunohistochemistry (IHC) and for HER2 by silver in situ hybridization (SISH) or IHC. The HER2 status was predominantly relying on the SISH data but for those cases in which SISH failed HER2 status was based on the IHC data and cases were considered HER2 positive if the IHC score was 3+, using the HercepTest. For Tn-C slides from 1 to 3 original tumour blocks were immunostained in the Ventana automated Immunohistochemistry (IHC) System (Ventana Benchmark XT and Ultra) using Ventana Ultra View DAB (760-500). IHC was conducted according to established protocols. Positive and negative controls were included in all staining runs. The primary antibody Tenascin-C DAKO, clone TN2, dilution 1 : 50 was used. The staining intensity of Tn-C was assessed in the periductal area and judged as negative/normal = 0, weak = 1, moderate = 2, or intense = 3, as earlier presented in [3]. We quantified the degree of the concentration of ducts, lymphocyte infiltration, and periductal fibrosis as 0, 1, or 2, as per criteria for scoring presented in [3], using up to five representative original H&E slides. Tn-C staining and the neoductgenesis criteria were scored by one pathologist (TS). A case with a combined score of 4 to 6 points was denominated as DCIS with neoductgenesis. In our earlier paper [3], we denominated cases with a total score of 5 or 6 as neoductgenesis but, in this cohort, we ended up with too few cases (*n* = 17) and therefore modified our criteria.

### 2.3. Mammographic Classification

Mammographic features were reclassified into seven groups, modified from Tabar et al. [1]: (1) a stellate lesion without associated calcifications, (2) a circular or oval mass without associated calcifications, (3) powdery calcifications with or without an associated tumour mass, (4) crushed stone-like (pleomorphic) calcifications with or without an associated tumour mass, (5) casting type calcifications with or without an associated tumour mass, (6) others, that is, galactographic findings, and (7) architectural distortion. A last group consisting of those with a normal mammogram was added to the analyses. Mammograms were reviewed by two independent radiologists (Tabar and SA), blinded to tumour biology and follow-up.

### 2.4. Statistical Analyses

The Chi-square test was used to compare the distribution between groups. Crushed stone-like calcifications with the largest number of patients were chosen as the reference group in the analyses of mammographic features. Mammographic classification data were analysed to test the consistency of two independent radiologists, respectively, using Fleiss' Kappa from rating scores. The interpretation of the Kappa results followed Shrout's instructions [7, 8]. Survival analyses were performed using Kaplan-Meier curves, including the Log-Rank test. Cox proportional hazards regression models were used to generate hazard ratios (HRs) with 95% confidence intervals (CIs). Stratification analyses were performed by type of surgery and RT. All statistical tests were two-sided, and *p*-values less than 0.05 were considered significant. Data were analysed using the SPSS Statistics, version 19 (IBM, Chicago, IL, USA), and SAS 9.3 (Cary, NC, USA). Primary endpoints were first ipsilateral breast cancer events (IBEs) divided into new in situ IBE or* invasive* IBE. We also analysed data for all invasive events (AIEs), including invasive IBE, regional recurrences, and generalized disease. We included contralateral invasive breast cancer and AIE in one separate analysis. DCIS patients with recurrences within three months from primary surgery were excluded.

## 3. Results

During the follow-up (mean 184.2 months), 107 IBEs were registered of which 53 were invasive IBE, 43 in situ IBE, and 11 an in situ IBE followed by an invasive IBE. There were 66 AIEs. Forty-eight invasive contralateral events were registered. One hundred and thirty four women died, 19 (4.1%) of them from breast cancer.

Baseline characteristics and histopathological data by mammography features are presented in Table 1. In 432 of the 458 cases the mammograms could be reviewed. Of all 432 mammograms, 89 (20.6%) were classified as showing casting type calcifications. The kappa-value between the two reviewers was 0.66 (95% CI 0.57–0.76) when dividing the mammograms as showing casting type calcifications or not. The casting type calcifications, as well as the other types of calcifications, were more often detected by mammography screening, compared to the other mammographic features. We could not see that casting type calcifications on the mammogram related to a higher proportion of mastectomies being performed or more adjuvant RT given after breast conserving surgery (BCS). Casting type calcifications were related to a higher nuclear grade, ER-negativity, PR-negativity, and HER2 overexpression. The casting type calcifications were not related to neoductgenesis defined by our three histopathological criteria or to Tn-C expression (Table 1).

According to our definition of histopathological neoductgenesis, 44 (9.8%) of 450 evaluable cases showed a total sum of four to six points. However, only seven of the 89 cases with casting type calcifications were classified as showing neoductgenesis (Table 2). Only three of those seven had five or six points as in our earlier definition of neoductgenesis. The 44 cases classified as neoductgenesis were highly related to a higher grade, ER-negativity, PR-negativity, and HER2 overexpression and also to higher proliferation and a high expression of Tn-C.

Regarding prognosis we analysed the entire cohort and those undergoing BCS separately. Casting type calcifications were related to a nonstatistically significant lower risk of IBE when analysing the whole cohort, HR 0.59 (CI 95% 0.32–1.07), and for women undergoing BCS, HR 0.65 (CI 95% 0.35–1.20) (Tables 3(a) and 3(b)). When looking at risk for in situ IBE and invasive IBE separately for women undergoing BCS, the risk of an in situ IBE among those with casting type calcifications was almost the same as for the reference group with crushed stone-like calcifications, HR 0.90 (CI 95% 0.41–1.95), while for invasive IBE the HR was 0.38 (CI 95% 0.13–1.08) (Table 3(a)). The risk of AIE in cases with casting type calcifications was lower but it was only statistically significant in the univariate analysis of the entire cohort HR 0.41 (CI 95% 0.17–0.99). Including contralateral invasive cancer did not change the results much, neither in the entire cohort nor among patients treated with BCS (data not shown).

We could not find any statistically significant differences regarding prognosis for cases with the histological criteria for neoductgenesis (Table 4); however, the results were similar as for cases showing casting type microcalcifications. There was a nonsignificant lower risk of invasive IBE and a nonsignificant higher risk of in situ IBE, HR 0.82 (CI 95% 0.29–2.27) and HR 1.60 (CI 95% 0.75–3.39), respectively. We could not see any relation between Tn-C expression and prognosis in our cohort (data not shown). Analyses for AIE including contralateral invasive events did show similar results for neoductgenesis and Tn-C compared to AIE only. When looking at generalized disease only (*n* = 28) among those with a mammographic classification, we could not detect any significantly worse prognosis for cases showing casting type calcifications (4 of 89; 5%) compared to others (24 of 343; 7%).

There were only seven women who had both casting type calcifications and a DCIS showing histological neoductgenesis and only four of these seven showed all three criteria, that is, casting type calcifications, DCIS with neoductgenesis, and a high expression of Tn-C. Even if the numbers were small, survival analyses were done and no statistically significant differences were observed. Two of the seven cases with casting type calcifications and DCIS with neoductgenesis developed an in situ IBE. The remaining five were free of recurrence. The same two cases with an in situ IBE were the only cases with events among those four showing all three criteria (mammographic casting type calcifications, neoductgenesis histopathologically, and Tn-C overexpression).

We also correlated DCIS with casting type calcifications to HER2 and ER status. HER2+/ER− lesions were found in 36% of the cases with casting type calcifications, HER2−/ER− lesions in 10%, and ER+ (Luminal A-B) lesions in 53% of the cases. Corresponding numbers for DCIS with crushed stone calcifications were HER2+/ER− lesions in 18%, HER2−/ER− lesions in 7%, and ER+ lesions in 75% of the cases and for DCIS with architectural distortion they were HER2+/ER− lesions in 8%, HER2−/ER− lesions in 19%, and ER+ lesions in 73%. DCIS with other types of mammographic features were HER2+/ER− in 10%, HER2−/ER− in 9%, and ER+ in 81% of the cases. The 44 DCIS showing histopathological neoductgenesis were HER2+/ER− lesions in 55% of the cases, HER2−/ER− in 10%, and ER+ in 35% of the cases while DCIS without signs of neoductgenesis were HER2+/ER− in 15%, HER2−/ER− in 9%, and ER+ in 76% of the cases. Five of the seven cases with both casting type calcifications and signs of neoductgenesis were HER2+/ER− lesions (71%) and no case was classified as HER2−/ER−. Three of the four cases with these criteria and Tn-C overexpression were HER2+/ER− (75%).

## 4. Discussion

Casting type calcifications could not be related to a worse prognosis in this study based on a population based cohort of women with pure DCIS and more than 15 years of follow-up. We found a lower risk of invasive events in cases with casting type calcifications and a nonsignificantly higher risk of new in situ IBEs.

Our aim was to include DCIS cases with mammographic casting type calcifications, histopathological signs of neoductgenesis, and high Tn-C expression in a model trying to identify lesions with a worse prognosis. The number of cases fulfilling these criteria in this cohort was low, only 4 of 458 cases. Both casting type calcifications and histopathological signs of neoductgenesis were related to a more aggressive tumour phenotype, that is, high grade, ER-negativity, PR-negativity, and HER2 overexpression. Unexpectedly, casting type calcifications and neoductgenesis did not correlate to each other and neither casting type calcifications nor neoductgenesis showed a worse prognosis.

Some earlier studies have reported on a relation between casting (or comedo) type calcifications and a worse prognosis in invasive breast cancer [1, 9] but results have been conflicting [10, 11]. Studies on prognosis for DCIS with casting type calcifications are sparse. Some studies have shown a relation between casting type calcifications and high grade and more extensive disease [12–16]. Also, a correlation with HER2 overexpression has been reported [17, 18]. So similar to our data, it seems like casting type calcifications are related to a more aggressive tumour phenotype. In an analysis of a subset of women in a randomized trial (SweDCIS) studying radiotherapy after BCS, casting type calcifications showed a nonstatistically significantly elevated relative risk of local recurrence, RR 2.1 (95% CI 0.9–4.8). But the risk was only increased for in situ IBE and not for invasive IBE [12]. An indication of the fact that DCIS with signs of neoductgenesis might have a poorer prognosis has been reported earlier by Tot [19] but evidence is sparse and we could not verify this in this study.

Prognostic factors for DCIS are not as well established as for invasive breast cancer [20]. HER2 expression, for example, has been related to a higher risk of recurrence but it seems like it only relates to in situ IBE [6, 21]. In the present cohort, we have earlier shown that HER2-positive DCIS had a higher total risk of IBE but a statistically significantly lower risk of invasive events [6]. This lower risk for invasive IBE was not observed until after more than 10 years of follow-up. The findings from the SweDCIS study [12] regarding casting type calcifications showing an elevated risk of in situ IBE might be related to a correlation between casting type calcifications and HER2 expression. Furthermore, in a study of 266 primary DCIS with a known recurrence [22, 23], we have seen that primary DCIS lesions with a subsequent invasive IBE were more often ER-positive, HER2-negative, and EGFR-negative, compared to primary DCIS with a subsequent in situ IBE.

A high proportion of cases with casting type calcifications (36%) and an even higher proportion of cases showing signs of neoductgenesis (55%) were HER2-positive/ER-negative. In the group combining the two criteria the proportion of cases being HER2+/ER− was over 70%. HER2 overexpression and ER-negativity are well established bad prognostic factors in invasive cancer but we cannot explain why a phenotype like this did not correspond to a worse prognosis in DCIS. The risk of breast cancer death was very low in the cohort but we expected the number of recurrences to be high enough to discover a relation to tumour biology and microcalcifications. Our results raise questions on how the progression from in situ to invasive carcinoma is driven and we need to find other factors involved in the natural history of DCIS. In this cohort HER2 and ER status was not known when making treatment decisions but mammographic features might have had an implication on treatment choice.

## 5. Conclusion

Casting type calcifications could not be related to a worse prognosis in pure DCIS. We found a lower risk of invasive events and a nonsignificantly higher risk of new in situ IBEs. Both DCIS tumours with casting type calcifications on the mammograms and tumours with a histopathological picture of neoductgenesis were related to ER-negativity, PR-negativity, and HER2 overexpression but they were not related to each other. We cannot explain why a more aggressive phenotype of DCIS did not correspond to a worse prognosis. Further studies on how the progression from in situ to invasive carcinoma is driven are needed.

## Figures and Tables

**Table 1:** Baseline patient and tumour characteristics in a DCIS cohort diagnosed between 1986 and 2004, by mammographic features (*n* = 458).

Ductal carcinoma in situ	Mammographic features								*p* value^*∗*^
	Stellate	Circular or oval shaped	Powdery calcifications	Crushed stone calcifications	*Casting type calcifications*	Galactographic finding	Architectural distortion	Normal mammography	
Numbers at risk (*n* = 432)	16	47	29	192	*89*	15	28	16	
*Age (mean, years)*	64.3	64.2	52.0	55.6	*56.5*	55.9	65.4	56.4	0.36
*Detection mode*									
Screening	10 (62.5%)	27 (57.4%)	24 (82.8%)	173 (90.1%)	*82 (92.1%)*	1 (6.7%)	11 (39.3%)	3 (20.0%)	<0.001
Not screening	6 (37.5%)	20 (42.6%)	5 (17.2%)	19 (9.9%)	*7 (7.9%)*	14 (93.3%)	17 (60.7%)	12 (80.0%)	
*Type of surgery *									
BCS	14 (87.5%)	40 (85.1%)	25 (86.2%)	159 (83.2%)	*64 (71.9%)*	9 (60.0%)	18 (64.3%)	12 (75.0%)	0.06
Mastectomy	2 (12.5%)	7 (14.9%)	4 (13.8%)	32 (16.8%)	*25 (28.1%)*	6 (40.4%)	10 (35.7%)	4 (25.0%)	
*Radiotherapy after BCS *									
Yes	4 (28.6%)	14 (35.0%)	13 (52.0%)	73 (45.9%)	*34 (53.1%)*	5 (55.6%)	6 (33.3%)	4 (33.3%)	0.42
No	10 (71.4%)	26 (65.0%)	12 (48.0%)	86 (54.1%)	*30 (46.9%)*	4 (44.4%)	12 (66.7%)	8 (66.7%)	
*Nuclear grade *									
1	2 (12.5%)	9 (19.1%)	4 (13.8%)	16 (8.3%)	*3 (3.4%)*	0 (0%)	4 (14.3%)	1 (6.2%)	<0.001
2	6 (37.5%)	32 (68.1%)	14 (48.3%)	56 (29.2%)	*21 (23.6%)*	12 (80.0%)	13 (46.4%)	7 (43.8%)	
3	8 (50%)	6 (12.8%)	11 (37.9%)	120 (62.5%)	*65 (73.0%)*	3 (20.0%)	11 (39.3%)	8 (50%)	
*Neoductgenesis*									
0–3p	14 (93.3%)	45 (100%)	26 (89.7%)	160 (85.6%)	*82 (92.1%)*	13 (86.7%)	28 (100%)	13 (81.2%)	0.14
4–6p	1 (6.7%)	0 (0%)	3 (10.3%)	27 (14.4%)	*7 (7.9%)*	2 (13.3%)	0 (0%)	3 (18.8%)	
*ER*									
≥10%	11 (73.3%)	37 (88.1%)	23 (71.9%)	128 (74.4%)	*41 (52.6%)*	12 (85.7%)	19 (67.9%)	8 (53.3%)	<0.001
<10%	4 (26.7%)	5 (11.9%)	6 (28.1%)	44 (25.6%)	*37 (47.4%)*	2 (14.3%)	9 (32.1%)	7 (46.7%)	
*PR*									
≥10%	9 (60.0%)	29 (67.4%)	21 (72.4%)	85 (50%)	*17 (23.3%)*	8 (61.5%)	16 (61.5%)	5 (33.3%)	<0.001
<10%	6 (40.0%)	14 (32.6%)	8 (27.6%)	85 (50%)	*54 (76.7%)*	5 (38.5%)	10 (38.5%)	10 (66.7%)	
*HER2*									
positive	4 (28.6%)	1 (2.4%)	24 (85.7%)	63 (35.2%)	*41 (51.9%)*	3 (20.0%)	4 (15.4%)	6 (40.0%)	0.002
Normal	10 (71.4%)	41 (97.6%)	4 (14.3%)	113 (64.8%)	*38 (48.1%)*	12 (80.0%)	22 (84.6%)	9 (60.0%)	
*Proliferation*									
High	2 (15.4%)	10 (27.0%)	8 (34.8%)	64 (40.3%)	*25 (36.8%)*	0 (0%)	13 (48.1%)	7 (46.7%)	0.96
Low	11 (84.6%)	27 (73.0%)	15 (65.2%)	95 (59.7%)	*43 (63.2%)*	11 (100%)	14 (51.9%)	8 (53.3%)	
*Tenascin-C*									
High	2 (28.6%)	11 (34.4%)	3 (15.0%)	46 (36.2%)	*22 (36.7%)*	3 (30.0%)	12 (52.2%)	8 (72.7%)	0.01
Low	5 (71.4%)	21 (65.6%)	17 (85.0%)	81 (63. 8%)	*38 (63.3%)*	7 (70.0%)	11 (47.8%)	3 (27.3%)	

^*∗*^ *p* values are calculated for casting type calcification versus all other types of mammographic features together. Breast conserving surgery = BCS.

**Table 2:** The correlation between histopathological features of neoductgenesis and patient and tumour characteristics in 458 cases with DCIS. Eight cases could not be classified.

	Neoductgenesis^*∗*^		*p* value
	Yes (*n* = 44) Number (%)	No (*n* = 406) Number (%)	
Age at diagnosis (*n* = 450)			
≤55 years	25 (43.2)	174 (42.9)	0.08
>55 years	19 (56.8)	232 (57.1)	
Mammographic casting type calcifications (*n* = 424)			
Yes	7 (16.3)	82 (21.5)	0.42
No	36 (83.7)	299 (78.5)	
Nuclear grade (*n* = 450)			
1	2 (4.5)	37 (9.1)	<0.001
2	5 (11.4)	167 (41.1)	
3	37 (84.1)	202 (49.8)	
ER (*n* = 411)			
≥10%	15 (36.6)	273 (73.8)	<0.001
<10%	26 (63.4)	97 (26.2)	
PR (*n* = 403)			
≥10%	7 (18.4)	190 (52.1)	<0.001
<10%	31 (81.6)	175 (47.9)	
HER2 (*n* = 403)			
Positive	30 (69.8)	100 (27.8)	<0.001
Normal	13 (30.2)	260 (72.2)	
Ki67 (*n* = 367)			
High	27 (69.2)	104 (31.7)	<0.001
Low	12 (30.8)	224 (68.3)	
Tenascin-C (*n* = 304)			
High	20 (55.6)	92 (34.3)	0.01
Low	16 (44.4)	176 (65.7)	

^*∗*^Neoductgenesis was defined as a score of 4 to 6 points, combining the scores for concentration of ducts (0–2), lymphocytic infiltration (0–2), and periductal fibrosis (0–2).

**(a):** 

Ductal carcinoma in situ	Mammographic features							
	Stellate	Circular or oval shaped	Powdery calcifications	Crushed stone calcifications	*Casting type calcifications*	Galactographic finding	Architectural distortion	Normal mammography
Numbers at risk (*n* = 432)	16	47	29	192	*89*	15	28	16
*IBEs (in situ or invasive) n* = 107								
Univariate HR (95% CI)	1.10 (0.40–3.05)	0.94 (0.47–1.86)	0.99 (0.47–2.10)	Reference	*0.59 (0.32*–*1.07)*	1.28 (0.51–3.21)	1.04 (0.47–2.30)	1.08 (0.39–3.00)
^*∗*^Adjusted HR (95% CI)	0.92 (0.33–2.55)	0.88 (0.44–1.74)	0.98 (0.46–2.07)	Reference	*0.67 (0.37*–*1.22)*	1.90 (0.75–4.81)	1.27 (0.57–2.82)	1.07 (0.38–2.97)
*AIEs n* = 66								
Univariate HR (95% CI)	1.58 (0.48–5.21)	1.11 (0.49–2.53)	0.62 (0.19–2.03)	Reference	*0.41 (0.17*–*0.99)*	1.28 (0.39–4.19)	1.55 (0.64–3.74)	1.53 (0.47–5.01)
^*∗*^Adjusted HR (95% CI)	1.47 (0.45–4.87)	1.11 (0.48–2.55)	0.63 (0.19–2.08)	Reference	*0.47 (0.20*–*1.15)*	1.87 (0.56–6.23)	1.98 (0.81–4.83)	1.63 (0.49–5.36)

^*∗*^Adjusted for type of surgery and postoperative radiotherapy. BCS = breast conserving surgery; IBE = ipsilateral breast event; AIE = IBE, regional recurrence and generalized disease.

**(b):** 

Ductal carcinoma in situ	Mammographic features							
	Stellate	Circular or oval shaped	Powdery calcifications	Crushed stone calcifications	*Casting type calcifications*	Galactographic finding	Architectural distortion	Normal mammography
Numbers at risk (*n* = 341)	14	40	25	159	*64*	9	18	12
*IBEs (in situ or invasive) n* = 101								
Univariate HR (95% CI)	1.03 (0.37–2.87)	0.97 (0.49–1.92)	0.98 (0.46–2.07)	Reference	*0.65 (0.35*–*1.20)*	1.63 (0.58–4.52)	1.36 (0.58–3.19)	0.88 (0.27–2.82)
^*∗*^Adjusted HR (95% CI)	0.92 (0.33–2.56)	0.90 (0.45–1.78)	0.99 (0.47–2.10)	Reference	*0.66 (0.36*–*1.22)*	1.79 (0.64–4.98)	1.22 (0.52–2.86)	0.83 (0.26–2.67)
*Invasive IBEs n* = 51								
Univariate HR (95% CI)	1.63 (0.49–5.43)	0.78 (0.27–2.25)	0.74 (0.22–2.45)	Reference	*0.38 (0.13*–*1.08)*	1.75 (0.41–7.42)	1.87 (0.65–5.43)	1.19 (0.28–5.03)
^*∗*^Adjusted HR (95% CI)	1.47 (0.44–4.93)	0.72 (0.25–2.09)	0.76 (0.23–2.52)	Reference	*0.38 (0.13*–*1.09)*	2.03 (0.48–8.69)	1.64 (0.56–4.78)	1.08 (0.25–4.59)
*In situ IBEs n* = 50								
Univariate HR (95% CI)	0.55 (0.07–4.06)	1.15 (0.47–2.83)	1.29 (0.49–3.41)	Reference	*0.90 (0.41*–*1.95)*	1.78 (0.42–7.57)	0.97 (0.23–4.14)	0.61 (0.08–4.55)
^*∗*^Adjusted HR (95% CI)	0.49 (0.07–3.61)	1.02 (0.41–2.52)	1.28 (0.48–3.38)	Reference	*0.91 (0.42*–*1.97)*	1.93 (0.45–8.21)	0.84 (0.20–3.61)	0.54 (0.07–4.02)
*AIEs n* = 60								
Univariate HR (95% CI)	1.66 (0.50–5.48)	1.29 (0.56–2.97)	0.67 (0.20–2.21)	Reference	*0.44 (0.17*–*1.15)*	2.38 (0.72–7.88)	2.16 (0.83–5.63)	1.24 (0.29–5.22)
^*∗*^Adjusted HR (95% CI)	1.57 (0.47–5.22)	1.22 (0.53–2.82)	0.67 (0.20–2.23)	Reference	*0.45 (0.17*–*1.16)*	2.49 (0.75–8.28)	2.05 (0.78–5.37)	1.21 (0.29–5.10)

^*∗*^Adjusted for postoperative radiotherapy. IBE = ipsilateral breast event; AIE = IBE, regional recurrence and generalized disease.

**Table 4:** Cox regression analyses by histopathological features of neoductgenesis in primary DCIS (*n* = 458).

Ductal carcinoma in situ	Histopathological features of neoductgenesis, 0–6 points			
	All women (*n* = 450)		Women undergoing breast conserving surgery (*n* = 352)	
	0–3 points *n* = 406	4–6 points *n* = 44	0–3 points *n* = 318	4–6 points *n* = 34
*Events*				
*IBE (in situ or invasive) *	*n* = 107	*n* = 12	*n* = 89	*n* = 10
Univariate HR (95% CI)	Reference	1.19 (0.65–2.17)	Reference	1.01 (0.53–1.94)
^*∗*^Adjusted HR (95% CI)	Reference	1.22 (0.67–2.23)	Reference	1.06 (0.55–2.04)
*Invasive IBE *	*n* = 47	*n* = 4	*n* = 56	*n* = 3
Univariate HR (95% CI)	Reference	0.82 (0.29–2.27)	Reference	0.61 (0.19–1.96)
^*∗*^Adjusted HR (95% CI)	Reference	0.85 (0.30–2.35)	Reference	0.64 (0.20–2.08)
*In situ IBE *	*n* = 46	*n* = 8	*n* = 43	*n* = 7
Univariate HR (95% CI)	Reference	1.60 (0.75–3.39)	Reference	1.44 (0.65–3.19)
^*∗*^Adjusted HR (95% CI)	Reference	1.66 (0.78–3.51)	Reference	1.54 (0.69–3.43)
*AIE *	*n* = 59	*n* = 5	*n* = 55	*n* = 3
Univariate HR (95% CI)	Reference	0.74 (0.30–1.85)	Reference	0.47 (0.15–1.49)
^*∗*^Adjusted HR (95% CI)	Reference	0.78 (0.31–1.94)	Reference	0.49 (0.15–1.56)

^*∗*^Adjusted for type of surgery and postoperative radiotherapy. IBE = ipsilateral breast event; AIE = IBE, regional recurrence and generalized disease.

## Abbreviations

DCIS: Ductal breast carcinoma in situ
TN-C: Tenascin-C
ER: Estrogen receptor
PR: Progesterone receptor
HER2: Human epidermal growth factor receptor 2
TMA: Tissue microarray
IHC: Immunohistochemistry
CI: Confidence interval
BCS: Breast conserving surgery
RT: Radiotherapy
IBE: Ipsilateral breast event
AIEs: All invasive breast events.
